# The influence of microRNAs and poly(A) tail length on endogenous mRNA–protein complexes

**DOI:** 10.1186/s13059-017-1330-z

**Published:** 2017-10-31

**Authors:** Olivia S. Rissland, Alexander O. Subtelny, Miranda Wang, Andrew Lugowski, Beth Nicholson, John D. Laver, Sachdev S. Sidhu, Craig A. Smibert, Howard D. Lipshitz, David P. Bartel

**Affiliations:** 1 Whitehead Institute for Biomedical Research, Cambridge, MA 02142 USA; 2 Howard Hughes Medical Institute, Cambridge, MA 02142 USA; 3 Department of Biology, Massachusetts Institute of Technology, Cambridge, MA 02139 USA; 4 Molecular Medicine Program, The Hospital for Sick Children, Toronto, ON M5G 0A4 Canada; 5 Department of Molecular Genetics, University of Toronto, Toronto, ON M5S 1A8 Canada; 6 Present address: Department of Biochemistry and Molecular Genetics, University of Colorado School of Medicine, Aurora, CO 80045 USA; 7 Donnelly Centre, University of Toronto, Toronto, ON Canada; 8 Department of Biochemistry, University of Toronto, Toronto, ON M5S 1A8 Canada

**Keywords:** MicroRNAs, Poly(A) tail, mRNA–protein complexes

## Abstract

**Background:**

All mRNAs are bound in vivo by proteins to form mRNA–protein complexes (mRNPs), but changes in the composition of mRNPs during posttranscriptional regulation remain largely unexplored. Here, we have analyzed, on a transcriptome-wide scale, how microRNA-mediated repression modulates the associations of the core mRNP components eIF4E, eIF4G, and PABP and of the decay factor DDX6 in human cells.

**Results:**

Despite the transient nature of repressed intermediates, we detect significant changes in mRNP composition, marked by dissociation of eIF4G and PABP, and by recruitment of DDX6. Furthermore, although poly(A)-tail length has been considered critical in post-transcriptional regulation, differences in steady-state tail length explain little of the variation in either PABP association or mRNP organization more generally. Instead, relative occupancy of core components correlates best with gene expression.

**Conclusions:**

These results indicate that posttranscriptional regulatory factors, such as microRNAs, influence the associations of PABP and other core factors, and do so without substantially affecting steady-state tail length.

**Electronic supplementary material:**

The online version of this article (doi:10.1186/s13059-017-1330-z) contains supplementary material, which is available to authorized users.

## Background

Despite often being depicted as long, naked molecules, messenger RNAs (mRNAs) in the cell are bound by a host of factors to form mRNA–protein complexes (mRNPs) [1–4]. In humans, hundreds of RNA-binding proteins (RBPs) have been identified, and, speaking to the complexity of mRNP complexes, many RBPs can bind the same transcript simultaneously [5, 6]. Some factors recognize specific motifs, often in 3′ untranslated regions (3′ UTRs), to regulate gene expression, whereas others recognize features common to mRNAs, such as the cap and poly(A) tail, and thus in principle have the potential to bind most mRNAs. The proteins bound to a transcript are critical for controlling its fate, and alterations in an mRNP can have profound effects for gene expression, even if the underlying mRNA sequence remains unchanged. For instance, during nutrient deprivation, 4E-BPs can compete with the translation-initiation factor eIF4G for binding to eIF4E, thereby repressing the translation of mRNAs of pro-growth genes independently of any modifications in the corresponding transcripts [7].

mRNPs are dynamic, and their organization changes throughout the life cycle of an mRNA [1, 3, 4, 8]. For instance, upon entry into the cytoplasm, nuclear mRNP components are exchanged for their cytoplasmic counterparts or, as is often the case with the exon-junction complex, removed entirely [9–11]. Decay also changes mRNP organization. Typically this process has been viewed from an RNA-centric perspective, in which deadenylation stimulates 5′ decapping, which leads to 5′ → 3′ exonucleolytic degradation of the transcript body [12–14]. However, dissociation of proteins that normally bind and protect the 5′ and 3′ ends of the transcript is also important to the process, as illustrated by deadenylation-independent decapping in *pab1Δ* yeast strains, which lack the cytoplasmic poly(A)-binding protein (PABP; known as Pab1p in yeast) [15, 16]. Similarly, eIF4E, in addition to acting as a core translation-initiation factor, sterically blocks access of the decapping enzyme to the 5′ cap, and its dissociation is a necessary, although poorly understood, step in mRNA decay [17]. Finally, recruitment of decay enzymes, especially deadenylases, further alters the makeup of an mRNP and is often thought to be an important step in regulating transcript stability [14, 18–22]. Thus, as has become increasingly appreciated, an mRNP-based perspective is important for understanding the mechanistic bases of post-transcriptional regulatory pathways.

An important post-transcriptional pathway in animals is the microRNA (miRNA) pathway, which appears to influence nearly every biological process in humans [23]. In most developmental contexts, metazoan miRNAs repress gene expression by stimulating mRNA decay and, to a lesser extent, repressing translation initiation [24–27]. These small RNAs are bound by an Argonaute protein (AGO) to form a silencing complex that is directed to targets via base-pairing between the miRNA and sequences that are typically found in the 3′ UTRs of targets [28]. In animals, once bound, miRNAs stimulate decay of their targets through an adapter protein called TNRC6 (GW182 in flies), which in turn recruits deadenylase complexes [20–22, 29–31], and deadenylation ultimately feeds into the canonical 5′ → 3′ decay pathway [20, 30, 32–34]. Interestingly, there are specific developmental contexts, such as in the pre-gastrulation fish embryo, where deadenylation, rather than stimulating decapping, leads to robust translational repression [35, 36]. These differing regulatory outcomes reflect broader differences in the relationship between poly(A)-tail length and post-transcriptional gene regulation in the early embryo, as opposed to differences in the direct effects of miRNA-mediated repression [36].

Mechanistic understanding of miRNA-mediated regulation has come from a variety of approaches, including genetic, molecular, biochemical, and structural experiments. Initial experiments, involving either knocking down various decay enzymes or overexpressing dominant-negative versions, revealed the central role for TNRC6/GW182 and canonical mRNA decay enzymes, including cytoplasmic deadenylases (both the CCR4–NOT and Pan2–Pan3 complexes), the decapping enzyme, and the Xrn1 exonuclease [30, 32–34]. Supplementing these genetic approaches have been molecular approaches in which the effects of tethering a protein of interest, such as TNRC6 or 4E-T, to a reporter transcript have been determined [29, 31, 37–40]. In addition to probing the role of complete cofactors, tethering experiments have been used to dissect the role of specific subunits of the CCR4–NOT complex and to identify the regions and amino acids of proteins that are necessary for eliciting repressive effects [37, 41]. For instance, the C-terminal domains of TNRC6 and GW182 are sufficient, when tethered, to repress expression, and they do so through direct interactions with the cytoplasmic deadenylase complexes and PABP [31, 37, 38]. Complementing these in vivo experiments have been biochemical and structural studies, which have illuminated a dense network of protein–protein interactions required to recruit the decay machinery during miRNA-mediated repression [42]. One recent example has been work on CNOT1, a scaffold protein in the CCR4–NOT deadenylase complex, which contains a mIF4G domain that interacts directly with DDX6/Me31B and is required to mediate repression of reporters [40, 41, 43, 44]. DDX6, as well as its orthologs, then interacts with decapping activators, such as 4E-T and Edc3, thereby bridging 3′ poly(A)-tail shortening with the eventual 5′ decapping [39, 45, 46].

Understanding the fate of proteins, such as PABP and eIF4E, during miRNA-mediated repression has been more challenging, and studies have primarily relied on reporter transcripts to characterize their dynamics. For instance, in vitro experiments in *Drosophila* lysates indicate that PABP dissociates during miRNA-mediated repression, that the dissociation of PABP works through GW182, and that this dissociation occurs prior to, rather than following, deadenylation [47]. Consistent with this result, PABP dissociation from reporters in the presence of the cognate miRNA is also observed in S2 cells, even when deadenylation has been blocked [38]. Similarly, in support of early studies that implicated eIF4F dissociation in translational repression [48, 49], work in both *Drosophila* and human cells demonstrates that components of the eIF4F complex (made up of eIF4E, eIF4G, and eIF4A) dissociate, although the specific factor that dissociates has varied depending on the experimental conditions [38, 50, 51]. Although each of these studies has provided important steps forward for understanding the ways in which mRNP organization can change during miRNA-mediated decay, the ramifications for endogenous mRNPs have been inferred based on a few reporter transcripts.

Direct characterization of endogenous target complexes typically relies on immunoprecipitation-based techniques, such as RNP immunoprecipitation (RIP), in which RNAs that co-purify with a protein of interest are identified. RIP-based approaches have long been recognized as powerful tools for characterizing mRNPs [52], and the advent of transcriptome-wide technologies has further expanded their utility. For instance, RIPs have been used to identify targets of a variety of regulatory factors, such as human HuR and *Drosophila* Smaug, Pumilio, and Brain tumor [53–56]. Similarly, RIP experiments have also been used to detect recruitment of Argonaute to its targets [57–60].

Despite their potential, RIP-based approaches have not been broadly applied to characterizing mRNP reorganization during miRNA-mediated regulation, especially with respect to dissociation of core factors from endogenous transcripts. Here we have applied the RIP-based approaches to eIF4E, eIF4G, PABP, and DDX6, each of which has been implicated as either dissociating or binding during miRNA-mediated repression. By using antibodies recognizing endogenous proteins, our investigations into mRNP reorganization were performed without any RBP overexpression. We further coupled this approach with transcriptome-wide measurements of poly(A)-tail length, in order to temporally link mRNP alterations with deadenylation. These datasets also allowed us to investigate mRNP organization more broadly and independently of miRNAs, which unexpectedly revealed that poly(A)-tail length explains little of the differences in mRNP organization observed between mRNAs of different genes in human cells, as well as in *Drosophila* S2 and *Saccharomyces cerevisiae* cells. Moreover, unlike steady-state poly(A)-tail length [36], binding of core factors, such as PABP, correlated with translation and stability, suggesting an overriding role of mRNP organization in control of gene expression. Together, our results emphasize the importance of an mRNP perspective not just for considering the mechanisms of miRNA-mediated repression but also for understanding post-transcriptional regulation more generally.

## Results

### Binding of eIF4G and PABPC1 to target mRNAs decreases during miRNA-mediated repression

To investigate how mRNP organization is altered during miRNA-mediated repression, we quantified the changes in mRNAs co-immunoprecipitating with key proteins of interest. In each experiment, HEK293 cells were transfected with either miR-124, miR-155, or no miRNA. After 24 h, RIP experiments were performed [52, 55, 56], and the change in mRNA abundance for each sample was then determined relative to the corresponding no-miRNA control. To minimize dissociation and reassociation of mRNA during the RIP procedure, the time of incubation with antibodies and beads was kept brief. Although the short incubation time reduced the overall yield of RNA, the protocol nonetheless maintained a > 40-fold enrichment of *GAPDH* mRNA in the eIF4G pull-down compared to the IgG control pull-down (Additional file 1: Figure S1).

**Fig. 1:**
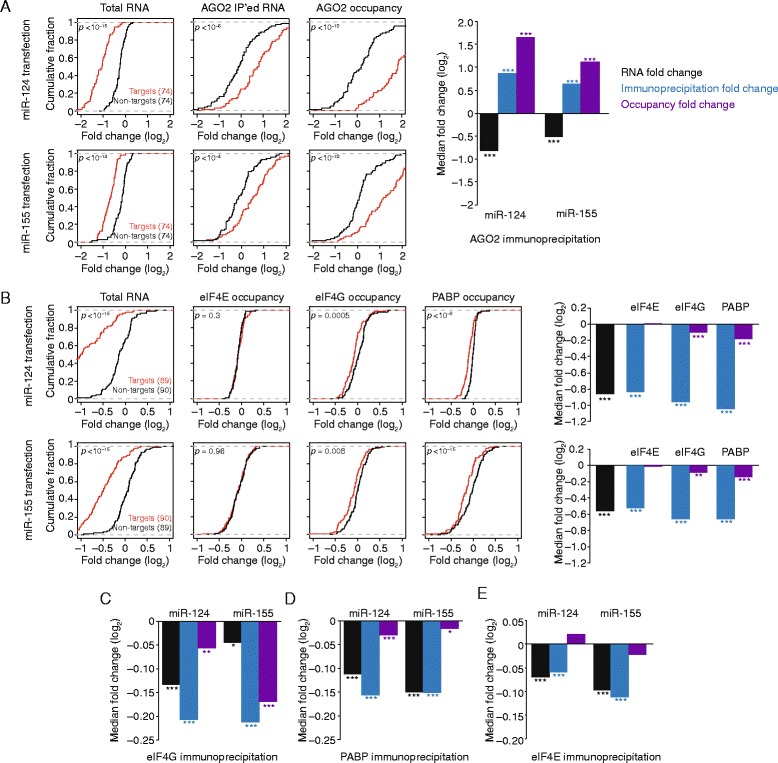
The influence of miRNAs on AGO2, eIF4E, eIF4G, and PABP occupancies on endogenous mRNAs. **a** The influence of miRNAs on mRNA abundance and AGO2 occupancy, as determined by RIP with NanoString quantification. Line graphs plot the cumulative distributions of changes in total mRNA (*left*), AGO2-immunoprecipitated mRNA (*middle*), and AGO2 occupancy (*right*) due to transfection of miR-124 (*top*) or miR-155 (*bottom*), distinguishing the results for targets of the transfected miRNA from those of the other miRNA (*red* and *black*, respectively). Bar plots show the median fold changes in total mRNA (*black*), AGO2-immunoprecipitated mRNA (*blue*), and inferred AGO2 occupancy (*purple*) attributable to the indicated miRNA. To arrive at these changes, median changes for non-target mRNAs were subtracted from those of target mRNAs. Significant differences in the cumulative distributions attributable to the miRNA are indicated: *p < 0.05; **p < 0.01; ***p < 0.0001; two-tailed K–S test. **b** The influence of miRNAs on mRNA abundance and eIF4E, eIF4G, and PABP occupancies. Otherwise, this panel is as in **a. c** The influence of miRNAs on eIF4G occupancy, as determined by RIP-seq. The effects on site-containing mRNAs were calculated relative to control mRNAs without sites. Otherwise, bar graphs are as in **a. d** The influence of miRNAs on PABP occupancy, as determined by RIP-seq and plotted as in **c. e** The influence of miRNAs on eIF4E occupancy, as determined by RIP-seq and plotted as in **c**

We first used the NanoString technology to quantify the RIP results for 193 unique mRNAs [53]. These included four highly abundant reference mRNAs (*ACTB*, *G6PD*, *GAPDH*, and *RPL19*) to allow intersample normalization, 95 high-confidence miR-124 targets, and 94 high-confidence miR-155 targets. These miRNA targets were chosen using the following two criteria: 1) robust repression following introduction of the cognate miRNA, as determined by quantitative mass spectrometry, global ribosome profiling, RNA sequencing (RNA-seq), and ribosome-footprint profiling [24–26]; and 2) the presence of at least one 7- or 8-nucleotide site to the cognate miRNA in the 3′ UTR and the absence of a seed-matched site to the non-cognate miRNA. We also ensured that the two target sets were indistinguishable across a variety of metrics, including 3′ UTR length, total length, and expression (Additional file 1: Figure S2), so that the miR-155 targets would serve as suitable no-site controls for the miR-124 transfections, and vice versa. As expected given the criteria used to choose the target sets, robust decreases in the RNA abundance of cognate targets was observed (Fig. 1a; median log_2_ fold change = −0.80, *p* < 10^−15^ for the miR-124 transfection; median log_2_ fold change = −0.52, *p* < 10^−15^ for the miR-155 transfection; two-tailed Kolmogorov–Smirnov [K–S] test).

To validate our approach, we first immunoprecipitated mRNPs using an antibody recognizing AGO2. Consistent with previous reports [58–60], targets were enriched in the AGO2 precipitations when cognate miRNA was introduced (Fig. 1a; median log_2_ fold change = 0.87, *p* < 10^−6^ for miR-124; median log_2_ fold change = 0.65, *p* < 10^−4^ for miR-155; K–S test). This increase in target mRNA binding to AGO2 occurred despite the decrease in target mRNA abundance. To account for this decreased abundance and thereby report the inferred change in AGO2 relative occupancy for the target mRNAs that remained after miRNA transfection, we normalized the change of each co-immunoprecipitated target mRNA to the change in its overall abundance. Note that although this normalization provided values for relative occupancy, not absolute occupancy, for simplicity, we refer to them hereafter as “occupancy” values. Overall, we observed robust increases in AGO2 occupancies for miRNA targets (Fig. 1a; median log_2_ fold change = 1.66, *p* < 10^−13^ for miR-124; median log_2_ fold change = 1.14, *p* < 10^−10^ for miR-155; K–S test). Together, these results, which were consistent with both the known role of AGO2 in miRNA-mediated repression [42] and the results of previous studies that used different precipitation and detection procedures [57–60], confirmed that our modified RIP protocol with NanoString quantification was informative.

We then performed analogous experiments quantifying mRNAs that co-immunoprecipitated with eIF4E, eIF4G, and PABP (Fig. 1b). In contrast to the strong increase in AGO2 occupancy, we observed a modest, but significant, decrease in eIF4G and PABP occupancy for target transcripts in the presence of the cognate miRNA (Fig. 1b). In the case of miR-124 transfection, eIF4G and PABP occupancy decreased by 7.3 and 11.7%, respectively (*p* = 0.0005 and *p* < 10^−8^, K–S test). For miR-155 transfections, eIF4G and PABP occupancy decreased by 5.4 and 8.4%, respectively (*p* = 0.008 and *p* < 10^−15^, K–S test). In contrast, no significant change in eIF4E occupancy was observed in response to either of the two miRNAs (*p* = 0.3 and *p* = 0.96, K–S test).

To check whether these results applied transcriptome-wide, we repeated the RIP experiment and analyzed RNA levels using RNA-seq. These RIP-seq experiments compared all predicted targets (mRNAs with at least one 7- or 8-nucleotide 3′ UTR site to the cognate miRNA) to control mRNAs that lacked a site to the cognate miRNA. As observed with NanoString detection, occupancies of both PABP and eIF4G, but not eIF4E, significantly decreased for predicted targets of the transfected miRNAs (Fig. 1c–e).

Our results with endogenous mammalian transcripts echoed observations of reduced eIF4G and PABP binding to reporters in *Drosophila* cells and extracts [38, 47] and supported the hypothesis that miRNA targeting promotes mRNP reorganization, such that binding of eIF4G and PABP is reduced—a trend we observed despite the vast diversity of mRNPs in the cell and their dynamic nature. Even though these reorganized mRNPs were also destabilized, they were sufficiently long-lived to be detected using steady-state analysis and thus likely represented the translationally repressed forms of the miRNA targets. Consistent with this idea, the magnitude of the reduced eIF4G and PABP binding resembled that observed for translational repression in HEK293 cells [27]. By similar reasoning, our inability to detect mRNPs lacking eIF4E, which must dissociate from the cap for decapping to occur, suggests that once eIF4E has dissociated from these mRNPs, miRNA-mediated repression triggers rapid decapping and degradation of the transcript body.

### DDX6 is recruited during miRNA-mediated repression

**Fig. 2:**
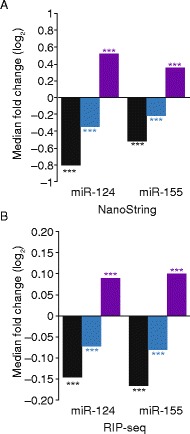
The influence of miRNAs on DDX6 occupancy on endogenous mRNAs. **a** The influence of miRNAs on mRNA abundance and DDX6 occupancy, as determined by RIP with NanoString quantification. Bar plots show the median fold changes in total mRNA (*black*), DDX6-immunoprecipiated mRNA (*blue*), and inferred DDX6 occupancy (*purple*) attributable to the indicated miRNA. Significant differences in the cumulative distributions attributable to the miRNA are indicated: *p < 0.05; **p < 0.01; ***p < 0.0001; two-tailed K–S test. **b** The influence of miRNAs on DDX6 occupancy, as determined by RIP-seq and plotted as in **a**

We next examined whether miRNA targeting increased binding of the decay factor DDX6. Although preferential binding of DDX6 to miRNA targets has not been directly demonstrated, this factor, which is thought to recruit the decapping enzyme [61], is required for miRNA-mediated repression and associates with CNOT1 of the CCR4–NOT complex through a structurally defined interface [41, 43, 44]. NanoString quantification of mRNAs that co-immunoprecipitated with DDX6 showed that miRNA targeting substantially increased DDX6 occupancy, with median increases of 43 and 27% observed for cognate targets after transfection of miR-124 and miR-155, respectively (Fig. 2a; *p* < 10^−10^ and *p* < 10^−6^, K–S test).

To examine the recruitment of DDX6 transcriptome-wide, we performed RIP-seq analysis. Because DDX6 associates with deadenylase complexes [41, 43, 44], RIP-seq libraries were prepared using ribosomal RNA depletion rather than poly(A) selection, reasoning that poly(A) selection would bias against deadenylated intermediates. (We note that because NanoString quantifies mRNA directly, without selection or amplification, our NanoString results were not confounded by differential enrichment of transcripts with differing poly(A) tail lengths.) As with the NanoString analysis, DDX6 occupancy on site-containing RNPs significantly increased in the presence of the cognate miRNA (Fig. 2b; median log_2_ fold change = 0.09, *p* < 10^−15^ for miR-124; median log_2_ fold change = 0.10, *p* < 10^−15^ for miR-155, K–S test). Together, these experiments demonstrate DDX6 enrichment in miRNA-targeted mRNAs, as expected if DDX6 recruitment is part of the mRNP reorganization that occurs during miRNA-mediated repression.

### DDX6 associates with mRNAs with shortened poly(A) tails

We noted that mRNAs from the vast majority of expressed genes passed our filtering in the DDX6 pull-down libraries. In pilot RT-qPCR experiments, *GAPDH* mRNA was enriched 28-fold in DDX6 pull-downs over control pull-downs (Additional file 1: Figure S3a). Normalizing RIP-seq results using this 28-fold enrichment suggested that transcripts from nearly all of the expressed genes were enriched above background in the DDX6 immunoprecipitations (Additional file 1: Figure S3b). Although we cannot formally rule out the idea that endogenous miRNAs down-regulate mRNA from nearly all of the expressed genes, this widespread DDX6 binding suggested that DDX6 plays roles in repressive post-transcriptional regulatory pathways in addition to the miRNA pathway, as well as in constitutive degradation. Such a model is consistent with its interaction with CNOT1, a core deadenylase component, and the observation that *DDX6* is an essential gene in many cell lines, including GBM cells, in which DICER is not essential [62].

**Fig. 3:**
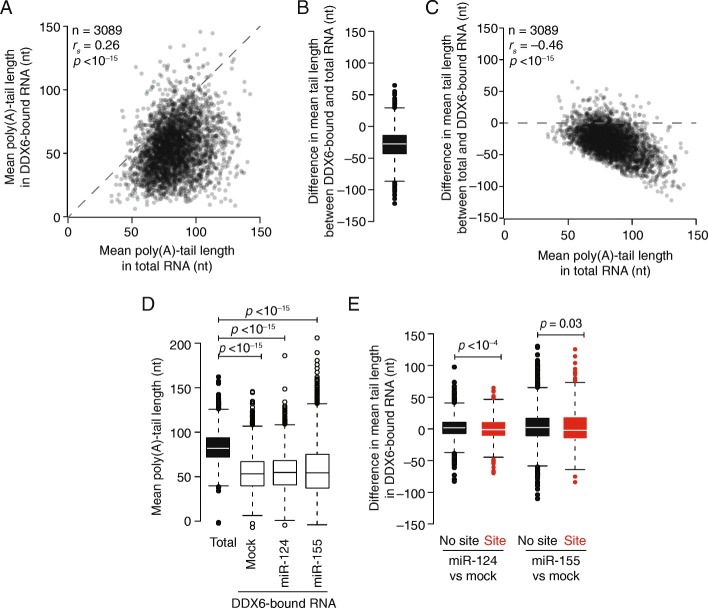
DDX6-bound mRNAs have shorter poly(A) tails. **a** Comparison of mean poly(A)-tail lengths of DDX6-bound mRNA with those of total mRNA. Results are plotted for each gene with sufficient PAL-seq tags in both samples. **b** The differences in mean poly(A)-tail lengths observed between DDX6-bound and total mRNA for each gene plotted in **a** (*white line*, median; *box*, quartiles; *whiskers*, 1.5 interquartile range). **c** Relationship between the difference in mean tail lengths observed between the DDX6-bound and total mRNA with respect to the mean tail lengths of total mRNA. **d** Mean poly(A)-tail lengths in the total mRNA and in DDX6-bound RNA following the indicated transfections (*line*, median; *box*, quartiles; *whiskers*, 1.5 interquartile range). Significance of the differences was evaluated using the paired Mann–Whitney U test. **e** The modest effects of miRNAs on mean poly(A)-tail lengths of DDX6-bound mRNAs. Plotted are the changes in mean poly(A)tail lengths of DDX6-bound mRNAs when comparing samples with the indicated transfected and mock-transfected miRNAs, distinguishing the results for mRNAs that contain at least one site to the cognate miRNA (site, *red*) from those for mRNAs that do not (no site, *black*). Significance was evaluated using the two-tailed K–S test

To explore the state of the DDX6-associated mRNA, we next investigated the relationship between DDX6 relative occupancy and poly(A)-tail length. We used poly(A)-tail length profiling by sequencing (PAL-seq) [36] to determine tail lengths in both DDX6-immunoprecipitated and steady-state total mRNA from mock and miR-124- and miR-155-transfected cells. When comparing results for the DDX6-immunoprecipitated mRNA with those of the total mRNA, mean tail lengths were weakly, although significantly, correlated (Fig. 3a; *r*_*s*_ = 0.26, *p* < 10^−15^). Strikingly, however, the mean tail lengths tended to be shorter in the co-immunoprecipitated samples (*p* < 10^−15^, Mann–Whitney U test). Indeed, for mRNAs from > 90% of the genes, mean tail lengths were shorter, with a median difference of 28 nucleotides (Fig. 3b). Our observation that mRNAs more associated with DDX6 have shorter tails than do co-expressed transcripts less associated with DDX6 is consistent with the direct interaction between DDX6 and the CCR4-NOT deadenylase complex [41, 43, 44].

Interestingly, mRNAs with the longest steady-state tail lengths also exhibited the largest amounts of deadenylation in the DDX6-bound population (Fig. 3c). This result is consistent with either of two models: DDX6 might preferentially associate with mRNAs with shortened tails, and thus relatively more of the poly(A) tail would need to be lost for DDX6 to associate with the initially long-tailed mRNAs. Indeed, as mentioned earlier, DDX6-bound poly(A)-tail length only weakly correlated with total-mRNA tail length (*r*_*s*_ = 0.26, *p* < 10^−15^), which is also consistent with a model in which DDX6 associates after some deadenylation has occurred. Alternatively, for longer-tailed species, DDX6 might associate at the onset of tail shortening and remain associated while deadenylation proceeds, before these transcripts are decapped and degraded.

We next determined the extent to which miRNA-mediated repression altered the tail lengths of DDX6-associated mRNAs. As observed for DDX6-bound mRNAs in the mock transfection, tails of DDX6-bound mRNAs in miR-124- and miR-155-transfected cells were significantly shorter than those of total mRNA in the cell (Fig. 3d; *p* < 10^−15^, Mann–Whitney U test). When comparing mRNAs with sites to the cognate miRNA to those without, median tail lengths of DDX6-bound mRNAs with sites were significantly shorter in the presence of the miRNA (Fig. 3e; *p* < 10^−4^ and *p* = 0.03, Mann–Whitney U test), although the magnitude of these differences (3–5 nucleotides) was small relative to the median amount of shortening undergone by the DDX6-bound population (27–29 nucleotides; Fig. 3d).

In summary, although DDX6 has been reported to be essential for miRNA-mediated repression, our results demonstrate that DDX6 is recruited to a broad diversity of mRNAs with short tails, thus arguing for roles of DDX6 beyond the miRNA pathway per se. Upon the addition of a miRNA, DDX6 was strongly recruited to the cognate targets, despite the fact that the tail lengths of the DDX6-bound cognate targets were not much shorter than those of other DDX6-bound mRNAs. This observation suggests that, when acting in the miRNA pathway, the dynamics of DDX6 binding are broadly similar to those of its binding in other mRNA decay pathways. Thus, the increased association of DDX6 with cognate targets is likely due to the recruitment of the CCR4–NOT complex (via TNRC6) rather than any direct interactions with machinery specific to the miRNA pathway, such as AGO2.

### PABPC1 dissociation tends to precede DDX6 recruitment and detectable miRNA-mediated deadenylation

**Fig. 4:**
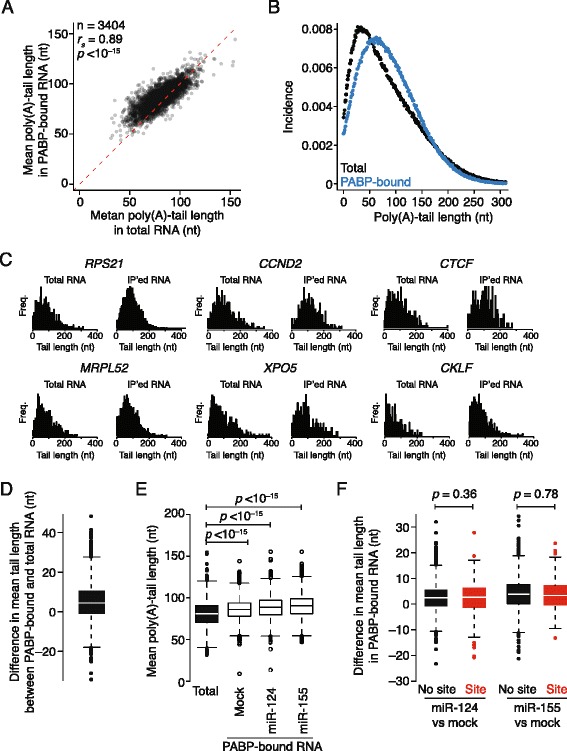
Poly(A)-tail lengths of PABP-bound transcripts. **a** Comparison of poly(A)-tail lengths of PABP-bound mRNA with those of total mRNA. Otherwise, this panel is as in Fig. 3a. **b** Tail-length distributions of metagenes constructed from total mRNA (*black*) and PABP-associated mRNA (*blue*). **c** Tail-length distributions of total mRNA and PABP-associated mRNA from the indicated genes. **d** The differences in mean poly(A)-tail lengths observed between PABP-bound and total mRNA for each gene plotted in **a** (*line*, median; *box*, quartiles; *whiskers*, 1.5 interquartile range). **e** Mean poly(A)-tail lengths in the total mRNA and in PABP-bound RNA following the indicated transfections. Otherwise, this panel is as in Fig. 3d. **f** The negligible effects of miRNAs on mean poly(A)-tail lengths of PABP-bound RNA. Otherwise, this panel is as in Fig. 3e

Work with reporters in vitro and in vivo has indicated that, during miRNA-mediated repression, PABP dissociates prior to mRNA deadenylation [38, 47] and, indeed, early studies of deadenylation suggest that PABP might need to dissociate in order for deadenylases, especially the CCR4-NOT complex, to act on a poly(A) tail [63]. To investigate this process for endogenous mRNAs transcriptome-wide, we determined the lengths of poly(A) tails in the total and PABP-bound fractions in the presence and absence of miR-124 or miR-155, using an antibody against PABPC1, the most highly expressed PABP in the cytoplasm of HEK293 cells. When comparing the PABP-bound and total mRNA populations, mean tail lengths were highly correlated (Fig. 4a; *r*_*s*_ = 0.89), which was significantly different from what we observed in the DDX6 analysis (Fig. 3a; *p* < 10^−15^, Fisher’s r-to-z calculation). These results suggested extensive overlap between the PABP-bound and total mRNA populations, and less overlap between the DDX6-bound and total mRNA populations, consistent with a role for PABP throughout most of the lifetime of the cytoplasmic mRNA but a role for DDX6 only in its waning phases.

Metagene analyses comparing the PABP-bound and total samples revealed that short poly(A) tails (i.e., those shorter than 50 nucleotides) were significantly depleted in the PABP-bound samples (Fig. 4b; *p* < 10^−9^, Wilcoxon rank test). We then examined the poly(A)-tail length distributions for six specific mRNAs (Fig. 4c), chosen because their PABP occupancies spanned a tenfold range and because their steady-state mean poly(A)-tail lengths varied by > 30 nucleotides (Fig. 4c). Although with these genes, as for many others, the mRNA tail-length distributions were broadly similar, the immunoprecipitated samples appeared to be depleted for short-tailed species. Consistent with this result, the overall mean tail lengths tended to be longer in the PABP-bound fraction than in the total population (Fig. 4d, e; *p* < 10^−15^, Mann–Whitney U test), although these differences were less than those observed in the DDX6 tail-length analysis (Figs. 3b cf*.* 4d; median differences of –28 and +4 nucleotides, respectively).

Interestingly, the metagene comparison also revealed that the longest poly(A) tails (i.e., those longer than 180 nucleotides) were also significantly depleted in the PABP-bound sample (Fig. 4b; *p* < 10^−15^, Wilcoxon rank sum test), although this depletion was more subtle than that for short tails. This depletion is consistent with some of the long-tailed mRNAs being preferentially associated with nuclear PABP, as expected if they were either still in the nucleus or freshly exported to the cytoplasm [64].

To explore a potential link between miRNA-mediated loss of PABP (Fig. 1b, d) and poly(A) tail shortening, we measured the tail lengths of PABP-bound mRNAs following either miRNA or mock transfection and compared results for mRNAs containing cognate sites with those of mRNAs lacking cognate sites. For both the miR-124 and the miR-155 analyses, no significant difference was observed (Fig. 4f; *p* = 0.36 and 0.78, respectively). Together, these results suggested that, as observed with reporters [38, 47], PABP might dissociate prior to or at the onset of miRNA-mediated deadenylation. The striking differences with the results for the DDX6 pull-downs, with respect to both the longer lengths of the tails of PABP-associated mRNAs and the lack of any detectable miRNA-mediated shortening of PABP-associated mRNAs, suggest that PABP tends to dissociate before DDX6 dissociates and, indeed, might even dissociate before DDX6 is recruited.

### Poly(A)-tail length exerts little influence on PABPC1 occupancy in human cells

The loss or recruitment of factors such as PABP and DDX6 during miRNA-mediated repression suggests that mRNP organization might vary for different mRNA species as a result of the cumulative effects of different regulatory pathways acting on these mRNPs. To explore mRNP organization more generally, we examined eIF4E, eIF4G, and PABP occupancies in untransfected cells.

Based on the enrichment of our benchmarking mRNA, *GAPDH*, essentially all expressed mRNAs were bound by these proteins, albeit to differing extents (Additional file 1: Figures S1 and S4). To examine directly whether lowly bound transcripts were indeed enriched in PABP immunoprecipitations, we used RT-qPCR to measure the amount of RNA in PABP pull-downs compared with control IgG pull-downs. We examined seven transcripts, which were chosen to represent a wide range of PABP occupancies (*GAPDH*, *SELK*, *RPLP2*, *ATP5I*, *CDKN3*, *MPRIP*, and *XIST*), and each of these transcripts was enriched in the PABP immunoprecipitations (Additional file 1: Figure S4a). Even *XIST*, whose occupancy was the lowest among those measured, was enriched in PABP immunoprecipitations. (Presumably, some *XIST* RNA, which is restricted to the nucleus, is bound by the small amount of cytoplasmic PABP found therein [65].) Moreover, the fraction of input immunoprecipitated by RT-qPCR corresponded with the RIP-seq occupancy values (*r*_*s*_ = 0.86; Additional file 1: Figure S4b). Together, these experiments indicate that even the lowest-bound mRNAs were nonetheless enriched in our PABP immunoprecipitations and that our RIP-seq occupancy values reflected quantitative differences in PABP binding.

**Fig. 5:**
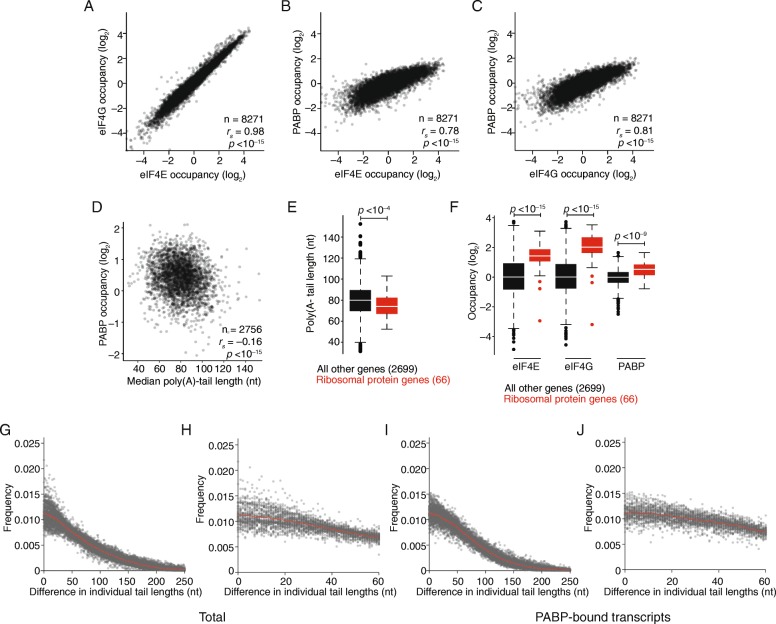
The weak relationship between mean poly(A) tail length and PABP occupancy in human cells. **a** Comparison of eIF4E and eIF4G occupancies for mRNAs of each gene that exceeded the expression cutoffs for quantification. **b** Comparison of eIF4E and PABP occupancies; otherwise as in **a. c** Comparison of eIF4G and PABP occupancies; otherwise as in **a. d** Relationship between PABP occupancy and mean poly(A)-tail length for mRNAs of each gene that exceeded the expression cutoffs for quantification. **e** Mean poly(A)-tail lengths of mRNAs of ribosomal protein genes (*red*) and of mRNAs of other genes (*black*). Significance was evaluated using the two-tailed K–S test. **f** eIF4E, eIF4G, and PABP occupancies for mRNAs of ribosomal protein genes (*red*) and those of other genes exceeding the expression cutoff (*black*). Significance was evaluated using the two-tailed K–S test. **g** Phasing analysis of poly(A)-tail lengths of total mRNA. For each of 100 randomly selected genes, each pairwise difference of individual tail lengths was calculated, and the normalized frequency for each possible difference was plotted (*grey points*) together with the average frequency (*red points*). **h** A zoomed-in version of **g**, focusing on differences between 0 and 60 nucleotides. **i** Phasing analysis of poly(A)-tail lengths of PABP-bound mRNA; otherwise, as in **g. j** A zoomed-in version of **i**, focusing on differences between 0 and 60 nucleotides

We next compared eIF4E and eIF4G occupancies and found that these were strongly correlated (Fig. 5a; *r*_*s*_ = 0.98, *p* < 10^−15^). Although this result was consistent with previous biochemical results, the correlation of eIF4E and eIF4G occupancies was stronger than might have been anticipated. Similarly, both occupancies were significantly correlated with PABP occupancy, although these relationships were not as strong as that observed between eIF4E and eIF4G (Fig. 5b, c; *r*_*s*_ = 0.78, *p* < 10^−15^ and *r*_*s*_ = 0.81, *p* < 10^−15^, respectively). Together, these results were consistent with the closed-loop model and the known interactions between eIF4G and both eIF4E and PABP [66, 67].

Although the core factors are usually considered to bind to nearly all cytoplasmic mRNAs, we observed a 10- to > 100-fold range in relative occupancies when comparing mRNAs from different genes (Fig. 5a–c). In the case of PABP, in vitro work has shown that PABP binds cooperatively and tightly to poly(A) stretches, leading to the prevailing idea that differences in poly(A) tail length cause corresponding differences in PABP binding. A hint of such a relationship was observed for mRNAs derived from the same gene, although when comparing PABP-associated and total mRNA the median difference between average tail lengths was only six nucleotides (Fig. 4d). However, when analyzing mRNAs from different genes we found that tail length did not positively correlate with PABP occupancy (*r*_*s*_ = –0.16; Fig. 5d). Similar results were observed with respect to eIF4E and eIF4G occupancy (*r*_*s*_ = –0.02, –0.04; Additional file 1: Figure S5). These results thus suggest that, in this human cell line, poly(A)-tail length has surprisingly little influence on mRNP organization, with no sign of a relationship between longer tails and greater occupancy of the factors examined. Indeed, mRNAs of ribosomal protein genes, which are known to have poly(A) tails that tend to be shorter than those of other genes (Fig. 5e; *p* = 0.001) [36], had some of the highest eIF4E, eIF4G, and PABP occupancies and, as a class, had occupancies that were significantly higher than those observed for the mRNAs of the rest of the genes (Fig. 5f; *p* < 10^−15^, *p* < 10^−15^, *p* < 10^−9^, respectively).

We next tested for evidence of phasing of poly(A)-tail lengths, as might be expected from cooperative binding of PABP dimers or oligomers on poly(A) tails [68], coupled with PABP-mediated protection of the tail from nucleases. We first examined the results for mRNAs of the individual genes we previously examined (Fig. 4b) but found no evidence of tail-length phasing when examining either total or PABP-bound transcripts (Additional file 1: Figure S6). Likewise, metagene analysis using all mRNA species with at least 100 poly(A) tail measurements also yielded no evidence of phasing (Fig. 5g–j). Perhaps cooperative PABP binding does not prevail in these cells. Alternatively, phasing might be obscured either by varied distance between the PABP dimer/oligomers and the start of the poly(A) tail or by varied numbers of tail nucleotides looped out between bound PABP monomers or even between the four RNA-binding domains of a single PABP molecule.

Taken together, our results indicate that, in human cells, longer-tailed transcripts from the same gene have somewhat higher PABP occupancy. However, differences in poly(A) tail-length do not explain the differences in PABP occupancy observed between mRNA species derived from different genes.

### Poly(A)-tail length exerts little influence on PABP occupancy in *Drosophila* and yeast cells

**Fig. 6:**
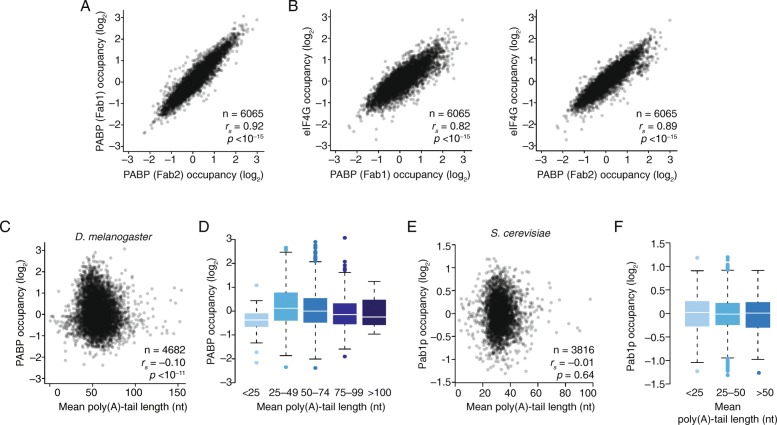
The weak relationship between PABP occupancy and mean poly(A)-tail length in *Drosophila* and yeast cells*.* **a** Comparison of PABP occupancies in *D. melanogaster* S2 cells determined using two different Fabs. Results are plotted for mRNAs from genes that exceeded the expression cutoffs for quantification. **b** Comparison of eIF4G occupancy with PABP occupancy, determined using either Fab1 or Fab2 (*left* and *right*, respectively). Otherwise this panel is as in **a. c** Relationship between PABP occupancy and mean poly(A)-tail length of mRNAs of *Drosophila* genes expressed in S2 cells. Otherwise, this panel is as in Fig. 5d. **d** PABP occupancies of mRNAs from S2 cells, grouping mRNAs by their mean poly(A)-tail length (*line*, median; *box*, quartiles; *whiskers*, 1.5 interquartile range). **e** Relationship between PABP occupancy and mean poly(A)-tail length of mRNAs of *S. cerevisiae* genes. Otherwise, this panel is as in **c. f** PABP occupancies of yeast mRNAs grouped by mean poly(A)-tail length. Otherwise, this panel is as in **d**

We next examined the relationship between steady-state poly(A) tail length and PABP binding in *Drosophila* S2 cells, using synthetic antibodies comprised of antigen-binding fragments (Fabs) developed against *Drosophila* PABP and eIF4G as part of a high-throughput platform focused on fly RNP components [69] (Additional file 1: Figure S7). Performing RIP-seq using two independent Fabs against PABP yielded highly correlated occupancy results (Fig. 6a; *r*_*s*_ = 0.92). In addition, PABP occupancy strongly correlated with eIF4G occupancy (Fig. 6b; *r*_*s*_ = 0.82–0.89), consistent with our results from human cells (Fig. 5c). Thus, the coupled binding of PABP and eIF4G to mRNAs appears to be a conserved phenomenon of vertebrate and invertebrate cells. We then compared PABP occupancy to published tail-length measurements from S2 cells [36]. As in human cells, there was no positive correlation between poly(A) tail length and PABP occupancy (Fig. 6c; *r*_*s*_ = −0.10). To explore this relationship further, genes were divided into bins based on their mean poly(A) tail length, using bins designed such that mRNAs of each successive bin would be able to accommodate about one additional PABP molecule. For instance, given the PABP footprint of 26 nucleotides [70], tails shorter than 25 nucleotides would be expected to bind at most one PABP, and tails between 25 and 49 nucleotides could accommodate up to two PABPs, and so on. Although, as expected, mRNAs from genes in the shortest-tail bin had significantly lower occupancy than those in the other bins (*p* < 10^−9^), a positive relationship between tail length and occupancy was not observed for the remaining bins (Fig. 6d).

We also examined this relationship in *S. cerevisiae*, making use of published datasets for both poly(A)-tail lengths and Pab1p binding [36, 71]. No correlation between steady-state poly(A)-tail length and Pab1p occupancy was observed in either the scatter-plot analysis (Fig. 6e, *r*_*s*_ = −0.01) or the binned analysis (Fig. 6f).

Thus, in cells from diverse eukaryotes—yeast, flies, humans—differences in steady-state poly(A)-tail length observed between genes cannot explain differences in PABP binding. Likewise, these tail-length differences cannot explain other RNP organizational differences that correlate with PABP occupancy, such as differences in eIF4G association.

### mRNP organization positively correlates with mRNA stability and translatability

**Fig. 7:**
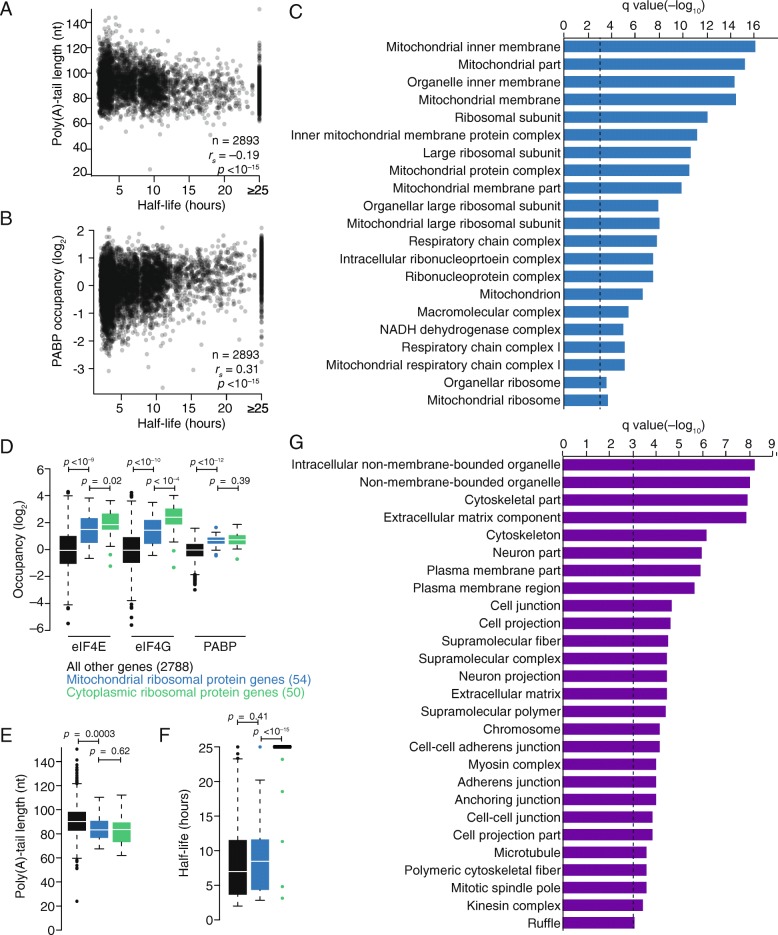
The relationship between PABP occupancy and transcript stability. **a** The relationship between mean poly(A)-tail length and mRNA half-life. Results are plotted for each gene that exceeded the expression cutoffs for quantification. **b** The relationship between PABP occupancy and mRNA half-life; otherwise as in **a. c** The top 21 gene ontology (GO) terms enriched in genes more unstable than predicted by their PABP occupancies, plotting for each term the log-transformed *q* value of its enrichment. *Dashed line* indicates a q-value of 0.001. **d** eIF4E, eIF4G, and PABP occupancies for mRNAs of mitochondrial ribosomal protein genes (*blue*), cytoplasmic ribosomal protein genes (*green*), and all other genes that exceeded the expression cutoffs for quantification (*black*) (*line*, median; *box*, quartiles; *whiskers*, 1.5 interquartile range). Significance was evaluated using the two-tailed K–S test. **e** Mean poly(A)-tail lengths of mRNAs of mitochondrial ribosomal protein genes, cytoplasmic ribosomal protein genes, and other genes; otherwise as in **d. f** mRNA stabilities of mRNAs of mitochondrial ribosomal protein genes, cytoplasmic ribosomal protein genes, and other genes; otherwise as in **d. g** The top 27 GO terms enriched in genes more stable than predicted by their PABP occupancies. Otherwise, this panel is as in **c**

Despite the known role for deadenylation in triggering mRNA decapping and decay, analyses of mRNAs from mammalian cell lines (HeLa and NIH3T3) have shown that mRNAs with longer steady-state tail lengths do not tend to be more stable [36]. Likewise, comparison of our steady-state poly(A)-tail length measurements with previously determined mRNA half-life measurements [72] did not show a positive correlation in HEK293 cells (Fig. 7a; *r*_*s*_ = −0.19). In contrast to tail length, PABP occupancy did correlate positively, albeit weakly, with mRNA stability (Fig. 7b; *r*_*s*_ = 0.31, *p* < 10^−15^). Similarly, PABP occupancy, and not poly(A)-tail length [36], correlated positively with translational efficiency previously determined using ribosome footprint profiling [27], although even less strongly than with stability (*r*_*s*_ = 0.16, *p* < 10^−15^). Consistent with PABP, eIF4E, and eIF4G coordinately binding to mRNAs, binding of eIF4E and eIF4G also correlated positively with mRNA stability and translational efficiency (*r*_*s*_ = 0.16 to 0.26, *p* < 10^−9^). Because these relationships are correlative and many mRNA features have co-evolved for optimal gene expression, ascribing causation is challenging. Nonetheless, our results support a model in which mRNP organization, involving binding of eIF4E, eIF4G, and PABP, is affected by regulatory pathways (such as that of miRNAs), in turn, affecting both mRNA stability and translation.

While performing these analyses, we noted substantial variation in the relationship between PABP binding and mRNA stability, which prompted examination of those genes with the largest deviations, focusing first on those that were less stable than predicted by their PABP occupancy. Surprisingly, this set of transcripts was highly enriched for those encoding proteins localized to the mitochondria. In fact, when gene ontology (GO) analysis was performed [73, 74], all of the top 21 GO terms were associated with mitochondrial complexes (Fig. 7c). Even those GO terms that at first appeared more general were driven by genes encoding mitochondrial proteins. For example, the enrichment for “ribosomal subunit” was not observed when we repeated this analysis after omitting mitochondrial ribosomal protein genes (Additional file 1: Figure S8).

To learn more about this result, features of mRNAs encoding mitochondrial ribosomal protein genes were investigated. As with mRNAs of cytoplasmic ribosomal proteins, mRNAs of their mitochondrial counterparts had a higher-than-average occupancy of eIF4E, eIF4G, and PABP (Fig. 7d; *p* < 10^−9^, *p* < 10^−10^, *p* < 10^−12^, respectively). These mRNAs also had short poly(A) tails, with lengths indistinguishable from those of mRNAs of the cytoplasmic ribosomal proteins (Fig. 7e; *p* = 0.62). However, these mRNAs were markedly less stable than those encoding cytoplasmic ribosomal proteins (Fig. 7f; *p* < 10^−15^; median half-life, 8.7 and 25 hours, respectively).

We next focused on mRNAs that were more stable than predicted by PABP binding and found that many of the associated GO terms described mRNAs with endoplasmic reticulum (ER)-associated translation, such as “extracellular matrix component” and “plasma membrane region” (Fig. 7g). Additional terms related to the cytoskeleton were also observed. Taken together, these results indicate that mRNAs with translation localized to the mitochondria and, perhaps, also the ER undergo regulation distinct from that of the general transcript population, especially with regard to mRNA decay.

## Discussion

### RIP and mRNPs

We used RIP in conjunction with either NanoString or high-throughput sequencing to examine endogenous mRNPs. One advantage of this approach is that it allows investigation of mRNAs and RNA-binding proteins expressed from their endogenous loci, and in so doing avoids over-expression of either the mRNA or protein components of mRNPs. Overexpression can lead to spurious binding and, thus, incorrect quantitative and qualitative definition of mRNP components relative to the endogenous situation in cells [75].

Despite these advantages, the RIP protocol includes in vitro incubation and washes that might perturb the native occupancies of some RNA-binding proteins. Although we modified the protocol to minimize the duration of the in vitro incubation, because we could not eliminate it altogether, the results that we report are for interactions that were sufficiently stable to survive these steps. Thus, any changes in the stability of interactions within an mRNP might have contributed, at least in part, to our observed changes in apparent occupancy. For example, apparent occupancy could have been inflated if remodeling of an mRNP added interactions that decreased the in vitro dissociation rate of the immunoprecipitated protein. Nonetheless, increasing the stability of an interaction often goes hand-in-hand with increasing its occupancy and, in either case, the result would reflect a change in the native mRNP. Overall, the correspondence between our results and those of previous approaches for studying miRNA-mediated regulation confirmed the utility of our approach, extended some of those previous results to endogenous mRNAs, and provided new insight into both miRNA-mediated repression and mRNP organization more generally.

### mRNPs in miRNA-mediated repression

Association with eIF4E, eIF4G, and PABP is a hallmark of stable, translationally competent cytoplasmic mRNPs, and a variety of reporter-based studies implicate miRNA-mediated repression in altering association with these key factors [38, 47, 50, 51]. With these results in mind, miRNA-dependent depletion of PABP-bound mRNA, measured using PABP RIP-seq, has been used to identify miRNA targets in human cells, although this was done without distinguishing depletion caused by lowered PABP occupancy from depletion caused by mRNA destabilization [55]. Our study has extended these results to endogenous mRNPs on a transcriptome-wide scale, showing that even after taking into account changes in RNA abundance the loss of PABP and eIF4G is widespread during miRNA-mediated repression, despite the inherently dynamic and diverse nature of mRNPs. The remodeled mRNPs that lost PABP and eIF4G were presumably incapable of supporting translation initiation, consistent with reports of the importance of the eIF4F complex in miRNA-mediated translational repression [49, 51].

Despite the readily detectable miRNA-mediated dissociation of PABP and eIF4G, we were unable to detect miRNA-mediated dissociation of eIF4E, which could be a consequence of miRNA-targeted mRNAs that have lost eIF4E undergoing decapping and degradation too rapidly to be detected by our methods. This interpretation is challenged by the results of our transcriptome-wide analysis of eIF4E binding, which revealed a range of eIF4E occupancies that spanned > 100-fold (Fig. 5a), implying that many mRNAs lacking bound eIF4E are not immediately decapped and degraded. We propose that the solution to this apparent paradox rests squarely on the mRNP context—that dissociation of eIF4E might have very different consequences for some mRNAs compared to others. In this model, eIF4E dissociation is necessary for decapping [17] but is not sufficient, and additional mRNP alterations associated with miRNA targeting would favor both decapping and degradation. Such alterations might include dissociation of PABP, shortening of the poly(A) tail, or recruitment of decay factors [4], each of which has been observed during miRNA-mediated repression in the present study (Figs. 1 and 2) as well as previous reports [30, 38, 47].

With respect to decay factors that predispose mRNAs for decapping, a top candidate is DDX6. This protein interacts with the decapping complex through adaptor proteins [39, 45] and is implicated in miRNA-mediated repression of reporters [41, 43, 44]. Moreover, we found that DDX6 is recruited to many endogenous mRNAs as they became targeted by miRNAs and that DDX6 is associated with many additional mRNAs undergoing decay, which presumably are not being targeted by miRNAs. DDX6-bound transcripts have poly(A) tails that are on average significantly shorter than those of the steady-state population, suggesting that DDX6 recruitment coincides with deadenylation. This result is consistent with recruitment of DDX6 by the CCR4–NOT deadenylase complex via a direct interaction with CNOT1 [41, 43, 44]. Nonetheless, our analysis of poly(A)-tail lengths on miRNA-site-containing mRNAs indicates that the downstream recruitment of DDX6 is broadly similar in the presence and absence of the cognate miRNA. Together, our results support a model in which miRNA-mediated repression leads to the recruitment of the CCR4–NOT deadenylase complex via direct interactions with TNRC6 [31, 37] and, once the deadenylase has been recruited, the dense network of interactions between mRNA decay factors then leads to the ultimate destruction of the transcript through mechanisms that also act on many other mRNAs and, thus, are not directly orchestrated by the miRNA machinery [42, 61].

### Organization of mRNPs: PABP and the poly(A) tail

PABP has long been recognized as a critical factor for post-transcriptional regulation and is essential for the ability of the poly(A) tail to stabilize transcripts. Consistent with this understanding, our results have demonstrated that increased PABP occupancy correlates with increased translatability and stability of the mRNA. The poly(A) tail has long been thought to be central in post-transcriptional regulation, with longer tails leading to increased stability and translation through their ability to bind more PABP. Unexpectedly, however, we found a lack of correlation between PABP occupancy and the length of the poly(A) tail, suggesting that the relationship between the poly(A)-tail length and PABP binding is more complex than previously thought. Indeed, although among transcripts derived from the same gene PABP occupancy was somewhat reduced for mRNAs with the shortest tails, differences in mean poly(A)-tail length could not explain differences in PABP occupancy observed for transcripts from different genes. Thus, although PABP might be critical for signaling that a poly(A) tail is present, in both human and fly cell lines and in yeast, PABP seems to be a very poor “reader” of poly(A)-tail length.

Recent studies have shown that, in contexts other than oocytes and early embryos, steady-state poly(A)-tail length fails to correlate with either mRNA stability or mRNA translation efficiency [36, 76]. These previous results can now be reconciled with the known roles of PABP in promoting mRNA stability and translation [15, 16, 77], in that we have shown here that differences in steady-state poly(A)-tail length do not necessarily cause differences in PABP occupancy.

Our results have also shown that the density of PABP along poly(A) tails can differ substantially for mRNAs from different genes. For instance, mRNAs for ribosomal proteins have very short poly(A) tails yet very high PABP occupancy. Although our current approach cannot determine the absolute number of PABPs bound to these mRNAs, our results are consistent with the density of PABP on these transcripts being higher than on other mRNAs. It will be interesting to explore this possibility further and to determine the extent to which PABP density influences deadenylation, decapping and/or other posttranscriptional processes.

What then might determine how much PABP is bound? Our results, together with those from *Drosophila* extracts [47], show that miRNA-mediated loss of PABP may just be the tip of the iceberg. The interplay among various regulatory factors, including miRNAs, and core factors, such as eIF4F, may determine the nature and composition of each mRNP, including the PABP occupancy of its constituent mRNAs. For instance, eIF4G can influence PABP affinity in vitro [78] and, thus, events in the 5′ UTR might have corresponding effects on PABP binding. This model is consistent with the numerous interactions described between PABP and other regulatory factors [22, 42, 66, 79]. Intrinsic mRNA features might also modulate PABP occupancy. For instance, because PABP can bind to AU-rich stretches with high affinity [80], differential binding to the 3′ end of the 3′ UTR, which can be quite AU-rich, might influence occupancy. Moreover, because PABP interacts with the termination factor eRF3, translation rate or termination efficiency might also have an impact on PABP binding [81]. Future insights into mRNP organization and remodeling will shed additional light on the mRNA features and factors that trump tail length to determine PABP occupancy.

## Conclusions

Here we have described the impact of miRNAs and poly(A) tail length on the association of core mRNP components and of the decay factor DDX6 in human cells. Extending previous reporter studies in *Drosophila* [38, 47], we show, for the first time, that miRNA-mediated repression in human cells is marked by the dissociation of eIF4G and PABP from, and the recruitment of DDX6 to, endogenous mRNPs. DDX6 is preferentially bound to transcripts with shortened poly(A) tails, suggesting that other repressive regulatory pathways, in addition to miRNA-mediated regulation, also recruit this factor. Moreover, although poly(A)-tail length has long been considered critical for the cytoplasmic fate of a transcript, differences in steady-state tail length explain little of the variation in PABP, eIF4E, or eIF4G association in human cells. Despite this finding, the relative occupancy of core components, including PABP, correlated with transcript stability and translation, confirming the importance of mRNP composition for gene expression. We thus propose a model in which post-transcriptional regulatory factors (such as miRNAs) alter the associations of PABP and other core factors without necessarily affecting steady-state tail length.

## Methods

### Cell culture

HEK293 cells (ATCC) were cultured as recommended by the manufacturer, in DMEM supplemented with 10% fetal bovine serum (Clontech) and penicillin/streptomycin. *Drosophila* S2 cells (Invitrogen) were cultured as described [82].

### Transfections

Cells were transfected with Lipofectamine 2000 (Invitrogen) and either 100 nM miRNA duplex or 5 μg pUC19 per five million cells, as recommended by the manufacturer. After 24 h, cells were harvested, and RNA was extracted using TRI-reagent (Life Technologies).

### RNA immunoprecipitations

EZ view protein G Sepharose (Sigma) was washed twice with lysis buffer A (100 mM KCl, 0.1 mM EDTA, 20 mM Hepes, pH 7.6, 0.4% NP-40, 10% glycerol, with freshly added 20 U/ml SUPERase•In [Ambion], 1 mM DTT and complete mini EDTA-free protease inhibitors [Roche; one tablet per 25 ml lysis buffer]). We used 100 μl slurry per five million cells. The beads were then blocked by an overnight incubation, rotating at 4 °C, with 500 μg salmon-sperm DNA (Sigma) and 1 ml buffer A. The next day, cells were washed with 1× PBS (137 mM NaCl, 2.7 mM KCl, 4.3 mM Na_2_HPO_4_, 1.47 mM KH_2_PO_4_, pH 7.4) and then lysed with 2 ml ice-cold buffer A per five million cells. After clarification by spinning at 15,000 × g for 10 minutes at 4 °C, lysates were incubated with antibodies for 1 h, rotating at 4 °C. Blocked Protein G beads were spun to remove the supernatant, resuspended in an equal volume of buffer A, added, and the lysates were further incubated for 1 h, rotating at 4 °C. The beads were washed three times with lysis buffer A, and then TRI-reagent was used to extract RNA. The AGO2 monoclonal 4G8 antibody was purchased from Wako Diagnostics; eIF4E and eIF4G1 antibodies were purchased from MBL international; PABPC1 and DDX6 antibodies were purchased from Abcam (ab21060 and ab40684, respectively). For Fab immunoprecipitations, control C1, anti-PABP (Fab1, D032; Fab2, D035) and anti-eIF4G (P190) Fabs were purified as described [69]. Anti-FLAG Sepharose (Sigma) was washed twice with lysis buffer A and then incubated overnight with the appropriate FLAG-tagged Fab and salmon-sperm DNA (Sigma), as above. Immunoprecipitations were performed as with standard antibodies, except that the Fab-bead conjugates were added directly to lysates and incubated for 2 h rotating at 4 °C.

### NanoString analysis

After RNA isolation, mRNA abundance was quantified using the NanoString nCounter system, according to the manufacturer’s instructions. Data were then analyzed using nSolver software and in-house scripts. For calculating occupancies using NanoString, we divided immunoprecipitation counts (normalized with NCounter) by input counts (normalized with NCounter).

### RNA-seq analysis

For quantifying relative levels of mRNAs using RNA-seq, mRNA TRU-seq libraries were prepared according the manufacturer’s directions (Illumina). In the case of DDX6 RIP-seq experiments, RNA was first depleted of ribosomal RNA using RiboZero (Illumina), according to the manufacturer’s instructions, and then Tru-seq libraries were prepared, starting with the reverse transcription step without any oligo(dT) selection and according to the manufacturer’s instructions. Libraries were pooled and sequenced on an Illumina HiSeq 2500 machine at The Whitehead Institute Genome Core or The Centre for Applied Genomics (The Hospital for Sick Children). Fifty-nucleotide single-end reads were demultiplexed and converted to FASTQ format using bcl2fastq2 v2.17 (Illumina). Library quality was inspected using FastQC v0.11.5 (http://www.bioinformatics.babraham.ac.uk/projects/fastqc/). Reads were mapped by STAR 2.5.2a [83] to the *Homo sapiens* (hg18) or *D. melanogaster* (dm6) genome. Genes were quantified using Cufflinks 2.2.1 [84], and gene expression was filtered for FPKM > 1 in both input and IP samples. In the case of miRNA experiments, genes were filtered for FPKM > 1 in both input and IP samples in the mock transfection, and non-zero FPKM in all other libraries. Relative occupancies were calculated for transcripts from each gene by dividing the IP FPKM by the input FPKM, and relative occupancies were median centered. The fold change in immunoprecipitation was calculated with values that were not normalized to input abundance. Downstream analyses were then performed with R version 3.3.1, using in-house scripts. Site-containing genes were defined as those with a 7mer or 8mer site in the 3′ UTR; no-site genes were those that had no seven- or eight-nucleotide site in the entire transcript and no six-nucleotide seed-matched site in the 3′ UTR.

### Tail-length measurements

After RNA isolation, PAL-seq libraries were prepared and analyzed as described [36]. Mean tail-length values were determined for all genes with ≥ 100 PAL-seq tags.

### RT-qPCR

After RNA extraction, input and immunoprecipitated RNA were treated with Turbo DNase (Thermo Fisher) and reverse transcribed with Superscript III (Invitrogen), according to the manufacturer’s instructions. For DDX6 immunoprecipitations, reverse transcription was primed with random hexamers. To amplify human transcripts with qPCR, the following primer sets were used: for *GAPDH*, AGCCTCAAGATCATCAGCAATG and CACGATACCAAAGTTGTCATGGAT; for *SELK*, TGCGCATTCATAGCAGAAGG and TCCCAGCATGACCTCATTCATC; for *RPLP2*, ATCTTGGACAGCGTGGGTATC and CTGGGCAATGACGTCTTCAATG; for *ATP5I*, ACCTAAAACCTCGGGCAGAAG and GGCAATCCGTTTCAGTTCATCC; for *CDKN3*, ACCCATCATCATCCAATCGC and AGGCAGGTTGTAAGCTCTTCC; for *MPRIP*, AAGAGGAGAAAGTGCACTGC and TGTGAAAGGTTTGCCACGAC; for *XIST*, AACACCCCTTTCTTCAGCTG and CCAGAAACTGTGAAAGGAAGGC. *Drosophila Actin5C* was amplified with AACACACCCGCCATGTATGT and ATTCCCAAGAACGAGGGCTG.

## Additional file

Additional file 1: Figure S1. Enrichment for *GAPDH* mRNA despite a shortened eIF4G RIP incubation. **Figure S2** Broadly similar characteristics of the miR-124 and miR-155 target sets detected by NanoString. **Figure S3.** DDX6 associates with mRNAs from most genes. **Figure S4** PABP binds to nearly all expressed mRNAs. **Figure S5.** Poor correlation between association of core translation factors and poly(A)-tail length. **Figure S6.** Phasing analysis of poly(A)-tail lengths of mRNAs from individual genes. **Figure S7.** Enrichment of *Act5C* mRNA in *Drosophila* PABP and eIF4G RIP-seq samples. **Figure S8.** The GO terms enriched in genes more unstable than predicted by their PABP occupancies, when performing the analysis after excluding genes for mitochondrial ribosomal proteins. (PDF 1012 kb)
