# Supplementary material for: The influence of microRNAs and poly(A) tail length on endogenous mRNA–protein complexes
Source: Genome Biol. 2017 Oct 31;18:211. doi: 10.1186/s13059-017-1330-z (PMC5664449; doi:10.1186/s13059-017-1330-z)
Supplement: Additional file 1: Figure S1. — Enrichment for GAPDH mRNA despite a shortened eIF4G RIP incubation. Figure S2 Broadly similar characteristics of the miR-124 and miR-155 target sets detected by NanoString. Figure S3. DDX6 associates with mRNAs from most genes. Figure S4 PABP binds to nearly all expressed mRNAs. Figure S5. Poor correlation between association of core translation factors and poly(A)-tail length. Figure S6. Phasing analysis of poly(A)-tail lengths of mRNAs from individual genes. Figure S7. Enrichment of Act5C mRNA in Drosophila PABP and eIF4G RIP-seq samples. Figure S8. The GO terms enriched in genes more unstable than predicted by their PABP occupancies, when performing the analysis after excluding genes for mitochondrial ribosomal proteins. (PDF 1012 kb) [file 13059_2017_1330_MOESM1_ESM.pdf]

ADDITIONAL FIGURE LEGENDS

Additional file 1: Figure S1. Enrichment for *GAPDH* mRNA despite a shortened eIF4G RIP incubation. The fraction of total *GAPDH* mRNA pulled down was determined by RT-qPCR. Plotted is the percent of the total pulled down by either a control antibody (anti-rabbit IgG, black) or anti-eIF4G after the indicated incubation times. The fold enrichment with anti-eIF4G antibody compared to the control antibody is indicated within each red bar.

Additional file 1: Figure S2. Broadly similar characteristics of the miR-124 and miR-155 target sets detected by NanoString. (A) Transcript lengths of miR-124 and miR-155 targets (black and red, respectively), based on Refseq annotations (line, median; box, quartiles; whiskers, range). Statistical significance was evaluated using the K-S test. (B) 3'UTR lengths of miR-124 and miR-155 targets; otherwise, as in (A). (C) 5'UTR lengths for miR-124 and miR-155 targets; otherwise, as in (A). (D) Expression levels of miR-124 and miR-155 targets, as determined by RNA-seq (Guo et al., 2010); otherwise, as in (A).

Additional file 1: Figure S3. DDX6 associates with mRNAs from most genes. (A) Enrichment of *GAPDH* mRNA in DDX6 immunoprecipitations. Otherwise this panel is as in Figure 1—figure supplement 1. (B) Distribution of DDX6 enrichment values transcriptome-wide, as determined by RIP-seq. For mRNAs of each gene that satisfied the expression cutoffs, DDX6 occupancy was calculated and then these relative

occupancy values were normalized by the enrichment of *GAPDH* in (A) to infer enrichment values.

Additional file 1: Figure S4. PABP binds to nearly all expressed mRNAs.

(A) Enrichment of transcripts in PABP immunoprecipitations in different biological replicates. RNA was immunoprecipitated with IgG or anti-PABP antibody. The percent of the indicated transcripts was quantified by RT-qPCR. Numbers reflect the fold enrichment of each transcript in PABP immunoprecipitations relative to the IgG control immunoprecipitation. (B) A comparison of PABP binding as determined by RT-qPCR and RNA-sequencing. Plotted is the PABP occupancy for the seven indicated genes, as measured by RIP-seq, and the percent of total RNA immunoprecipitated, as measured by RT-qPCR. RT-qPCR measurements were an average of the 3 independent, biological replicates from (A); error bars denote standard deviation.

Additional file 1: Figure S5. Poor correlation between association of core translation factors and poly(A)-tail length. (A) Relationship between eIF4E occupancy and mean poly(A)-tail length. Otherwise this panel is as in Figure 5D. (B) Relationship between eIF4G occupancy and mean poly(A)-tail length. Otherwise this panel is as in Figure 5D.

Additional file 1: Figure S6. Phasing analysis of poly(A)-tail lengths of mRNAs from individual genes. Pairwise differences of individual tail lengths were calculated and plotted (purple), together with a smoothened curve (blue), for both the steady-state and the PABP-associated samples.

Additional file 1: Figure S7. Enrichment of *Act5C* mRNA in Drosophila PABP and eIF4G RIP-seq samples. Plotted is the percent of total *Act5C* mRNA pulled down with control antibodies (anti-rabbit IgG and C1 fab) and anti-PABP and anti-eIF4G fab, as determined by RT-qPCR.

Additional file 1: Figure S8. The GO terms enriched in genes more unstable than predicted by their PABP occupancies, when performing the analysis after excluding genes for mitochondrial ribosomal proteins. Otherwise, this figure is as in Figure 7C.

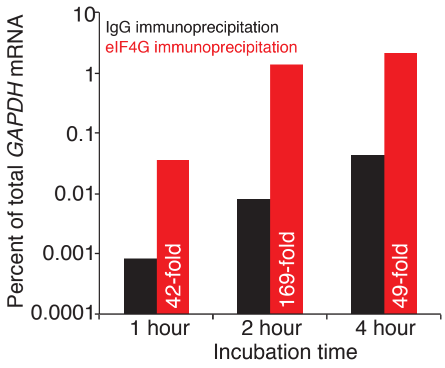

IgG immunoprecipitation
eIF4G immunoprecipitation
Percent of total GAPDH mRNA
10
1
0.1
0.01
0.001
0.0001
42-fold
169-fold
49-fold
1 hour
2 hour
4 hour
Incubation time

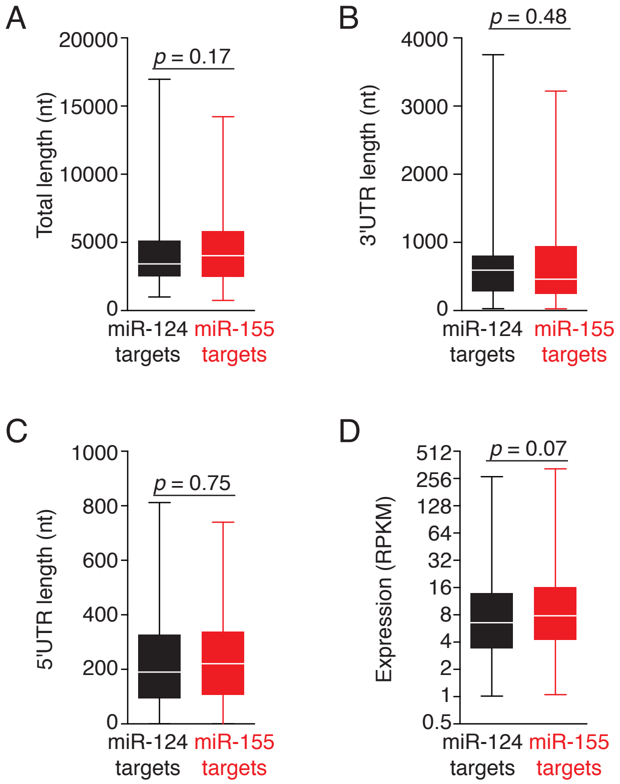
A
20000
15000
10000
5000
0
Total length (nt)
p = 0.17
miR-124 targets
miR-155 targets
B
4000
3000
2000
1000
0
3'UTR length (nt)
p = 0.48
miR-124 targets
miR-155 targets
C
1000
800
600
400
200
0
5'UTR length (nt)
p = 0.75
miR-124 targets
miR-155 targets
D
512
256
128
64
32
16
8
4
2
1
0.5
Expression (RPKM)
p = 0.07
miR-124 targets
miR-155 targets

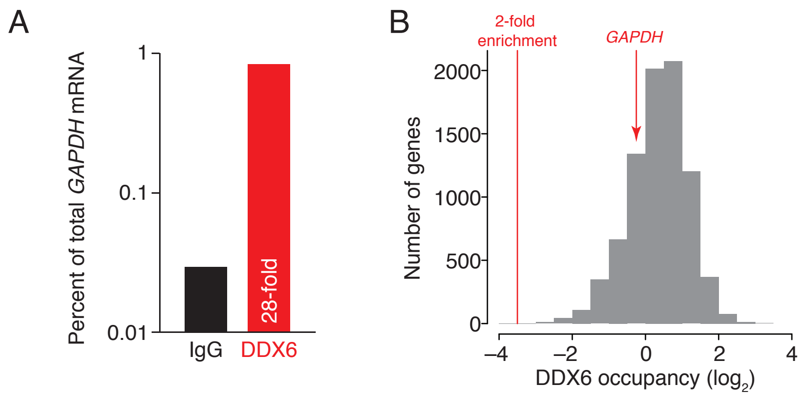
A
Percent of total GAPDH mRNA
1
0.1
0.01
28-fold
IgG
DDX6
B
2-fold
enrichment
GAPDH
Number of genes
2000
1500
1000
500
0
–4
–2
0
2
4
DDX6 occupancy (log2)

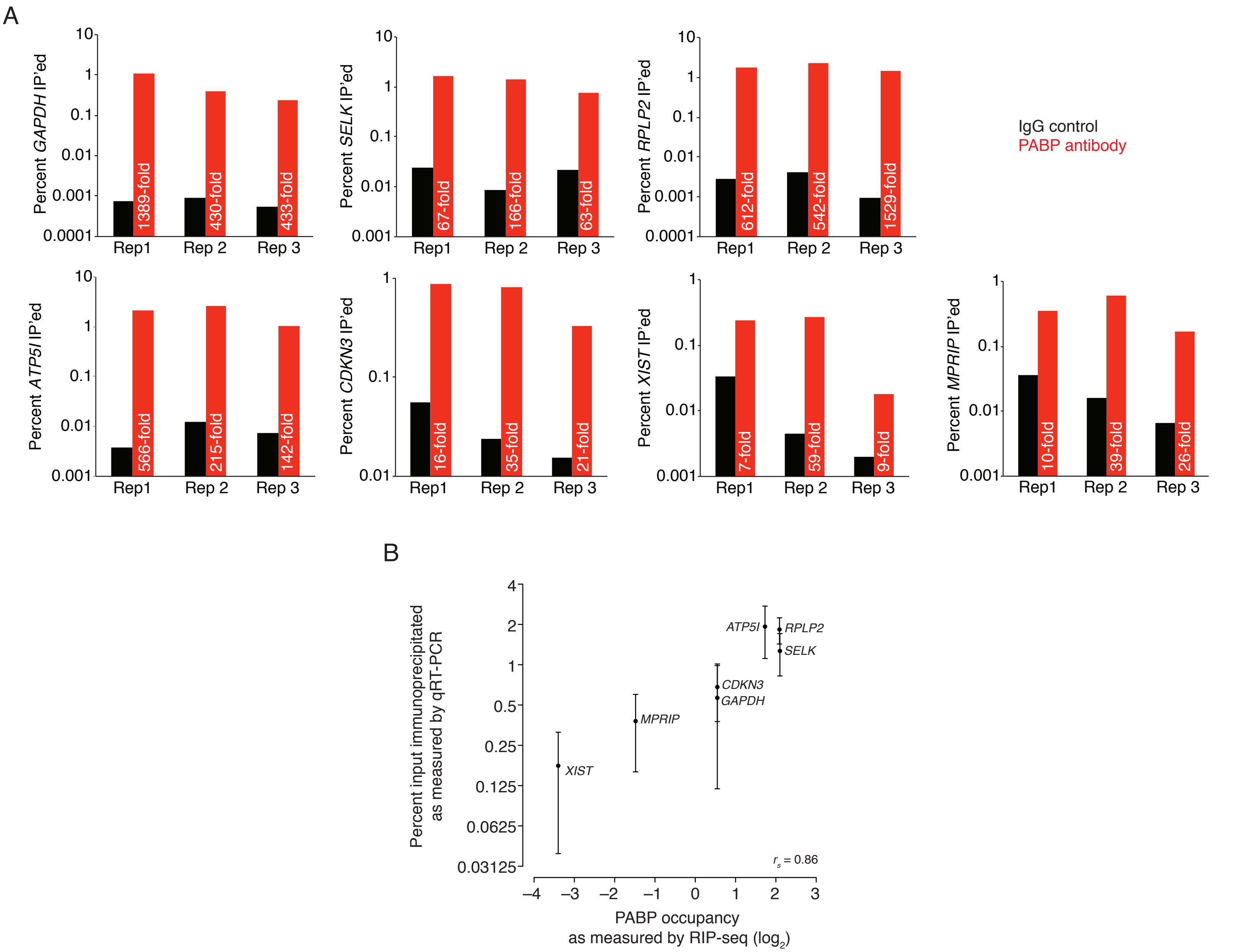
A
Percent GAPDH IP'ed
1389-fold
430-fold
433-fold
Percent SELK IP'ed
67-fold
166-fold
63-fold
Percent RPLP2 IP'ed
612-fold
542-fold
1529-fold
IgG control
PABP antibody
Percent ATP5I IP'ed
566-fold
215-fold
142-fold
Percent CDKN3 IP'ed
16-fold
35-fold
21-fold
Percent XIST IP'ed
7-fold
59-fold
9-fold
Percent MPRIP IP'ed
10-fold
39-fold
26-fold
Rep1
Rep 2
Rep 3
B
Percent input immunoprecipitated as measured by qRT-PCR
PABP occupancy as measured by RIP-seq (log2)
ATP5I
RPLP2
SELK
CDKN3
GAPDH
MPRIP
XIST
rs = 0.86

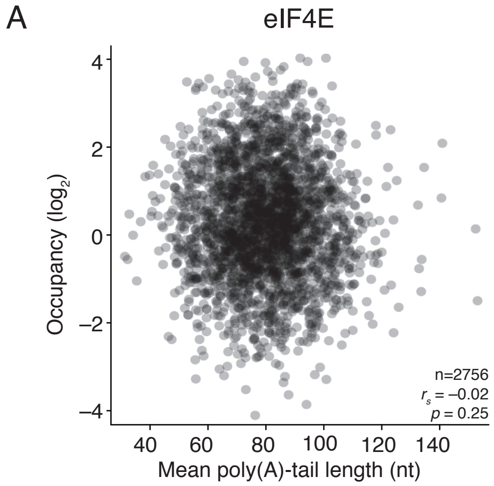
A
eIF4E
Occupancy (log2)
4
2
0
−2
−4
40
60
80
100
120
140
Mean poly(A)-tail length (nt)
n=2756
rs = −0.02
p = 0.25
B
eIF4G
Occupancy (log2)
4
2
0
−2
−4
40
60
80
100
120
140
Mean poly(A)-tail length (nt)
n=2756
rs = −0.04
p = 0.05

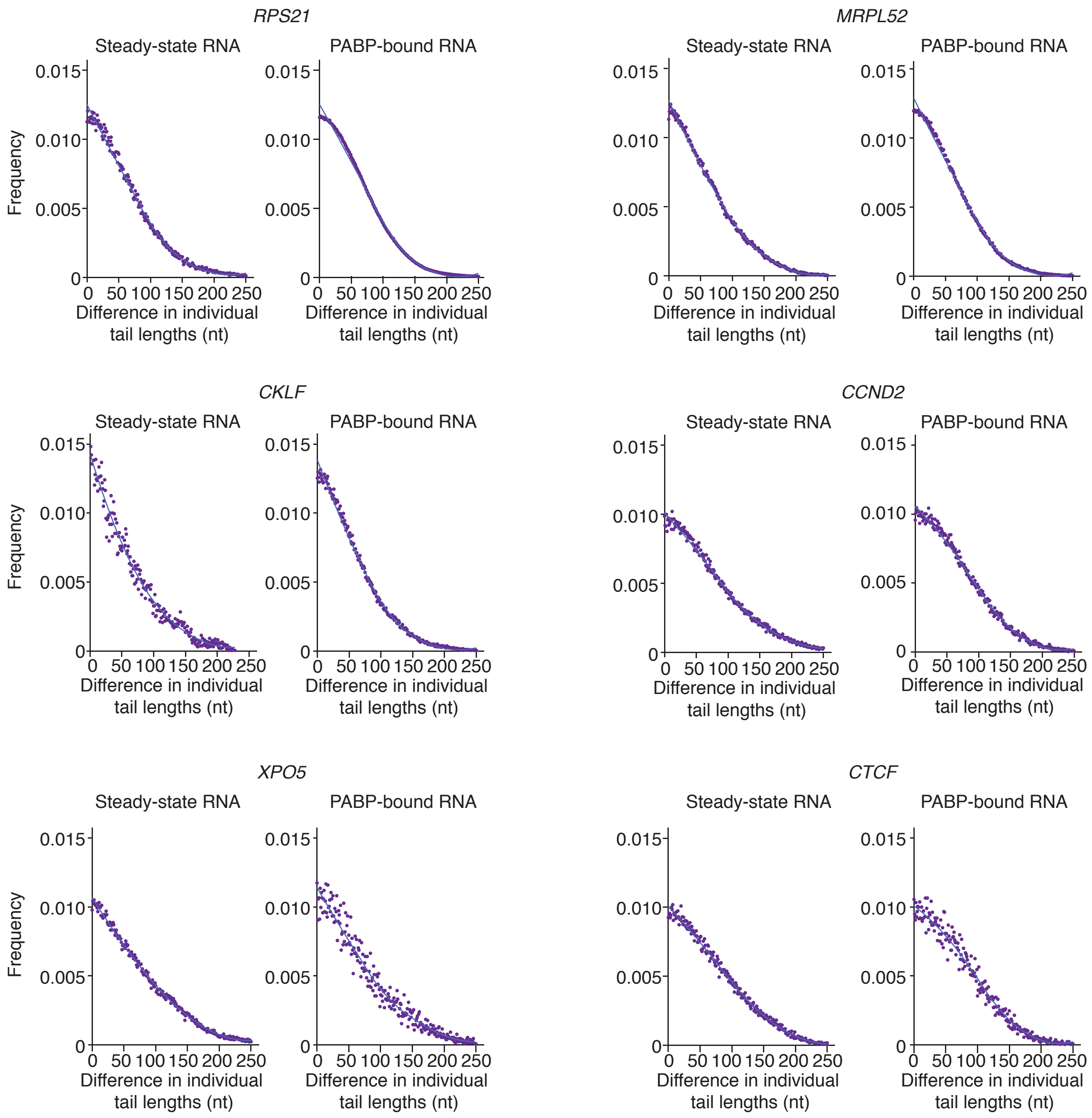
RPS21
Steady-state RNA
PABP-bound RNA
MRPL52
CKLF
CCND2
XPO5
CTCF
Frequency
0.015
0.010
0.005
0
0 50 100 150 200 250
Difference in individual tail lengths (nt)

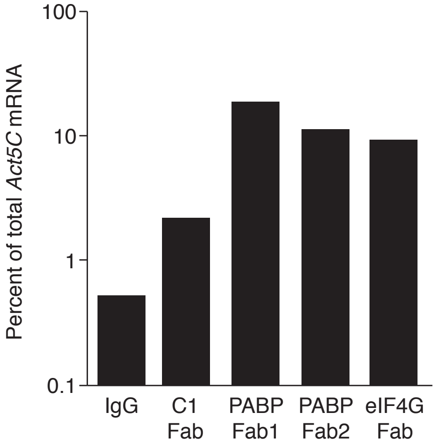
100
10
1
0.1
Percent of total Act5C mRNA
IgG
C1 Fab
PABP Fab1
PABP Fab2
eIF4G Fab

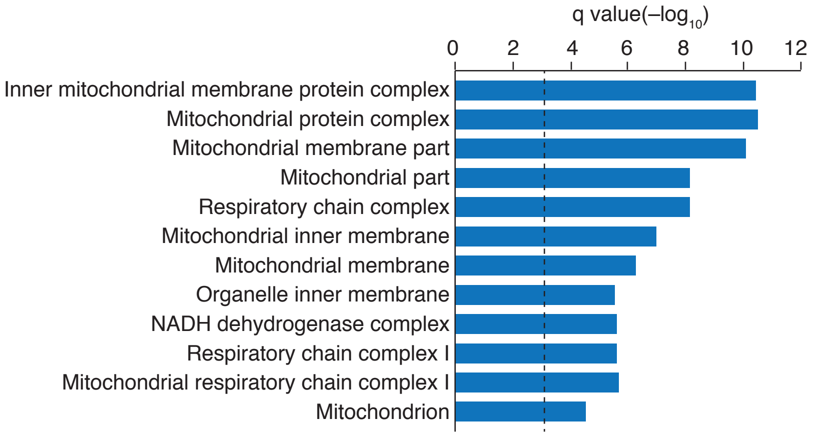
q value(–log10)
0
2
4
6
8
10
12
Inner mitochondrial membrane protein complex
Mitochondrial protein complex
Mitochondrial membrane part
Mitochondrial part
Respiratory chain complex
Mitochondrial inner membrane
Mitochondrial membrane
Organelle inner membrane
NADH dehydrogenase complex
Respiratory chain complex I
Mitochondrial respiratory chain complex I
Mitochondrion
